# Improved field emission stability from single-walled carbon nanotubes chemically attached to silicon

**DOI:** 10.1186/1556-276X-7-432

**Published:** 2012-08-01

**Authors:** Cameron J Shearer, Adam Fahy, Matthew Barr, Paul C Dastoor, Joseph G Shapter

**Affiliations:** 1 Institut für Physikalische Chemie, Westfälische Wilhelms-Universität Münster, Münster 48149, Germany; 2 Flinders Centre for Nanoscale Science and Technology, School of Chemical and Physical Sciences, Flinders University, Bedford Park, Adelaide 5042, Australia; 3 Centre for Organic Electronics, School of Physics, University of Newcastle, Callaghan, Newcastle, 2308, Australia

**Keywords:** Single-walled carbon nanotubes, Chemical attachment, Field emission, Field emission stability, Nanoelectronics

## Abstract

Here, we demonstrate the simple fabrication of a single-walled carbon nanotube (SWCNT) field emission electrode which shows excellent field emission characteristics and remarkable field emission stability without requiring posttreatment. Chemically functionalized SWCNTs were chemically attached to a silicon substrate. The chemical attachment led to vertical alignment of SWCNTs on the surface. Field emission sweeps and Fowler-Nordheim plots showed that the Si-SWCNT electrodes field emit with a low turn-on electric field of 1.5 V μm^−1^ and high electric field enhancement factor of 3,965. The Si-SWCNT electrodes were shown to maintain a current density of >740 μA cm^−2^ for 15 h with negligible change in applied voltage. The results indicate that adhesion strength between the SWCNTs and substrate is a much greater factor in field emission stability than previously reported.

## Background

Carbon nanotubes (CNTs) have a high aspect ratio and high electrical conductivity which make them prime candidates for field emission electrodes [1,2]. The practical application of a CNT-based field emission device requires both a low turn-on electric field (*E*_to_) and a stable output current [3]. Single-walled carbon nanotubes (SWCNTs) are accepted to have excellent field emission properties including a low turn-on field and high electric field enhancement factor (*β*) since their small diameter provides the highest aspect ratio compared with multi-walled carbon nanotubes (MWCNTs) [4-6]. Conversely, field emission from SWCNTs is usually regarded to be fragile because the single-shelled SWCNTs are less resilient to emission degradation mechanisms such as ion bombardment and Joule heating. Recently, much work has focused upon improving the field emission properties of MWCNTs due to their inherent emission stability [7,8]. Posttreatments to SWCNT films, such as plasma exposure, have been shown to significantly increase emission stability but at the cost of increasing *E*_to_[9]. Improving the emission stability from SWCNT electrodes without adversely affecting *E*_to_ and *β* is an ultimate goal in the field [3].

We have recently reported field emission from SWCNTs chemically attached to silicon and showed that these devices could withstand field emission current densities up to 500 μA cm^−2^ and were relatively stable, with the voltage required to maintain a current density of 95 μA cm^−2^ only increasing by 15% after 15 h and by 36% after 65 h [10]. More recently, we investigated field emission properties and stability from functionalized single-, double-, and multi-walled CNTs chemically attached to silicon where we found that the degree of functionalization played a major role in emission stability [11].

Recent experiments on Si-SWCNT electrodes have shown that a 2-h attachment time yields superior photovoltaic and electrochemical devices [12-14]. The field emission stability of 2-h Si-SWCNT electrodes has not been previously investigated. In this letter, we improve significantly upon previous Si-SWCNT electrodes and demonstrate that the chemical attachment of SWCNTs to a silicon substrate is a simple route toward the fabrication of a SWCNT electrode with stable emission while maintaining excellent values for *E*_to_ and *β*.

## Methods

Si-SWCNT electrodes were fabricated following the chemical attachment of functionalized SWCNTs as described in detail elsewhere [15]. Briefly, n-type highly antimony-doped Si wafers were cut to 0.25 cm^2^ and cleaned ultrasonically in acetone for 2 min. The Si wafers were then hydroxylated by stepwise immersion in 1:1:5 NH_4_OH:H_2_O_2_:H_2_O followed by HCl:H_2_O_2_:H_2_O for 20 min at 80°C. The wafers were then incubated for 2 h in a solution of dimethyl sulfoxide containing 0.2 mg mL^−1^ carboxylic acid-functionalized SWCNTs and dicyclohexylcarbodiimide [15]. The Si-SWCNT wafers were then washed ultrasonically for 2 min and dried in a stream of nitrogen. Field emission measurements were collected using a parallel plate setup with Si-SWCNT electrodes as the cathode and a stainless steel disk as the anode separated by 1.82 mm as determined by a micrometer screw [11]. All measurements were taken using a LabVIEW-controlled Keithley source measure unit (Keithley Instruments Inc., Cleveland, OH, USA). The base pressure of the field emission testing system was <1 × 10^−8^ Torr.

## Results and discussion

**Figure 1:**
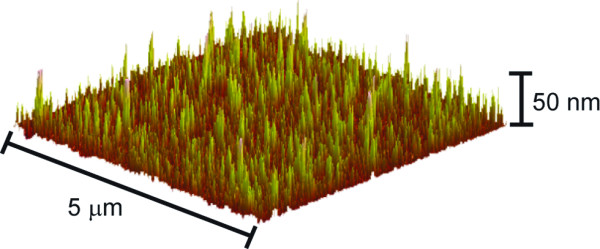
AFM image of SWCNTs vertically aligned on silicon.

Figure1 presents an atomic force microscopy (AFM) image of the prepared Si-SWCNT electrode. The SWCNTs form vertically aligned bundles, consistent with previous observations for SWCNTs chemically attached to substrates using this method [16-18]. Vertical alignment of the SWCNTs is due to two main effects: (a) the hydrophilic hydroxyl groups on the surface repel the hydrophobic walls of the SWCNTs, forcing the SWCNTs away from the surface; and (b) the high density of carboxylic acid groups on the ends of the SWCNTs promotes end-on surface attachment. The SWCNT bundle density is in the order of approximately 1 × 10^9^ cm^−2^ with heights of the features observed ranging from 20 to 100 nm with an average bundle diameter of 75 nm as determined by AFM analysis. 

**Figure 2:**
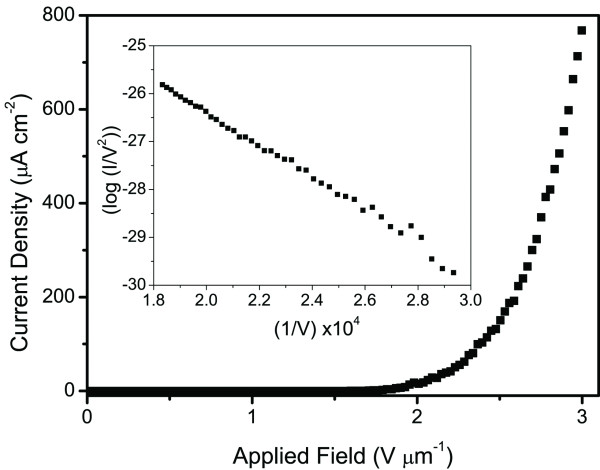
**Field emission *J *-*V *sweep from Si-SWCNT electrode with (inset) Fowler-Nordheim plot.**

A field emission *JV* sweep and its corresponding Fowler-Nordheim (F-N) plot for the Si-SWCNT electrode is shown in Figure2. The maximum field emission current density (*J*_max_) measured was 790 μA cm^−2^ and was limited by the maximum current that could be supplied by the field emission apparatus, indicating that *J*_max_ was actually higher than 790 μA cm^−2^. Field emission is demonstrated by the highly linear F-N plot [19], and from the slope of this plot, *β* was calculated to be 3,965 (assuming a SWCNT work function of 4.8 eV) [20]. In addition, these devices exhibited an *E*_to_ (electric field for *J* = 10 μA cm^−2^) and an *E*_th_ (electric field for *J* = 100 μA cm^−2^) of 1.5 V μm^−1^ and 2.37 V μm^−1^, respectively. 

The low *E*_to_ and high value for *β* demonstrate that the Si-SWCNT electrode is an elite field emission device [3,6,11,21,22]. The measured values are perhaps surprising given the short length and wide bundle diameter of the SWCNTs as determined by AFM. We hypothesize that by using a solution containing SWCNTs with a variety of lengths, we have produced a surface whereby the electrical field screening between SWNTs is minimal, leading to the excellent field emission characteristics observed [23]. Indeed, this hypothesis is supported by Chhowalla et al*.* who showed that short and stubby CNTs outperformed taller and thinner CNTs [24]. They argued that electrical field screening between adjacent CNTs was reduced when the CNT forest did not have a uniform height and the CNTs had a greater spacing.

**Figure 3:**
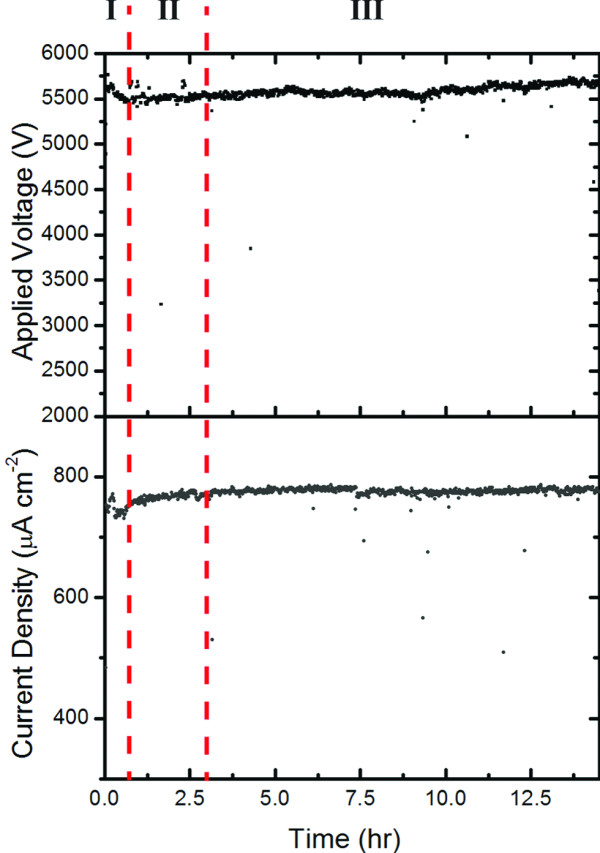
**Field emission stability of Si-SWCNT electrode.** Variation of both applied voltage (*V*-*t*) and current density (*J-t*) recorded with respect to time. Dashed red lines were added to signify the three regions of emission stability discussed in the text.

The emission stability from the Si-SWCNT electrode was tested by monitoring the voltage required to maintain a current of 740 μA cm^−2^ for 15 h. The variation of both the applied voltage (*V*-*t*) and the current density (*J*-*t*) as a function of time are presented in Figure3. There appears to be three main regions to the *V*-*t* and *J*-*t* plots:

1. *For t < 0.5 h*, the field emission is relatively unstable with both *V* and *J* fluctuating as a function of time, which is consistent with previous observations [25] and is most probably related to field-induced desorption of adsorbates, causing fluctuations in both the work function and *β*[26-28].

2. *For 0.5 < t < 2.5 h*, *V* is essentially constant while *J* increases to 770 μA cm^−2^ at *t* = 2.5 h. This observation is consistent with a decrease in sample resistance due to the removal of amorphous carbon or non-emitting SWCNTs, resulting in improved field emission [10].

3. *For 2.5 < t < 15*, *J* remains constant while *V* increases slowly from 5,500 V to approximately 5,700 V at *t* = 15 h, which is consistent with slow degradation of the field emission properties of the SWCNT film most likely due to ion bombardment and Joule heating processes [27-30].

A total voltage change of 1.8% over 15 h for an applied current of approximately 750 μA cm^−2^ is very low for a SWCNT field-emitting electrode. The only SWCNT electrode in the literature with greater stability was a screen-printed SWCNT electrode that was treated with an Xe/Ne plasma to improve stability to the point where a current density of 100 μA cm^−2^ was maintained for >50 h with minimal degradation [9]. However, the plasma treatment increased the *E*_to_ of the electrode from 2.9 V μm^−1^ to 4.3 V μm^−1^, effectively negating the advantages of using SWCNTs. Moreover, the output current density described here is over seven times greater with negligible field emission degradation observed.

The highly stable electron field emission at a relatively high *J* that is observed for the Si-SWCNT electrodes reported in this letter is attributed to a number of factors. First, the strong chemical attachment between the SWCNTs and the substrate will reduce the occurrence of field-induced CNT desorption, resulting in a more consistent field emission. Second, the bundling of the SWCNTs on the surface may also assist with the observed low degradation, with the outermost SWCNTs protecting the inner SWCNTs from ion bombardment. Third, as we have previously shown, the high crystallinity of these SWCNTs improves emission stability [11]. Finally, we propose that variation in the length of the emitting structures results in low electric field screening of the surface and a concomitant large population of emitting SWCNTs, leading to a high field emission current density [23].

## Conclusions

In summary, a field-emitting electrode consisting of SWCNTs chemically attached to a silicon substrate has been produced. The Si-SWCNT electrode was shown to field emit with an *E*_to_ of 1.5 V μm^−1^ and *β* of 3,965. The emission was shown to be remarkably stable with a current of approximately 750 μA cm^−2^ maintained for 15 h with a net voltage increase of only 1.8%. The chemical attachment of SWCNTs to Si is a simple, upscalable approach to produce SWCNT field emission electron sources with excellent characteristics and stability without the need for posttreatment.

## Competing interests

The authors declare that they have no competing interests.

## Authors' contributions

CJS prepared and characterized the Si-SWCNT electrodes and completed the field emission experiments with the assistance of AF, MB, and PCD using an apparatus maintained by AF, MB, and PCD. The experiments were conceived by JS, PCD, and CJS. All authors read and approved the final manuscript.

## Author details

^1^Institut für Physikalische Chemie, Westfälische Wilhelms-Universität Münster, Münster 48149, Germany. ^2^Flinders Centre for Nanoscale Science and Technology, School of Chemical and Physical Sciences, Flinders University, Bedford Park, Adelaide 5042, Australia. ^3^Centre for Organic Electronics, School of Physics, University of Newcastle, Callaghan, Newcastle 2308, Australia.
